# AFI manual planning versus HyperArc auto‐planning: A head‐to‐head comparison of SRS plan quality

**DOI:** 10.1002/acm2.14503

**Published:** 2024-09-05

**Authors:** Dharmin D. Desai, Ivan L. Cordrey, E. Lee Johnson, Thomas A. Oldland

**Affiliations:** ^1^ Varian Medical Systems Inc. Advanced Oncology Solutions Hixson Tennessee USA; ^2^ Thompson Cancer Survival Center Cumberland Medical Center Crossville Tennessee USA; ^3^ Department of Radiation Medicine University of Kentucky Chandler Medical Center Lexington Kentucky USA

**Keywords:** AFI, HyperArc, plan quality, R50%, SIMT, SRS

## Abstract

**Introduction:**

HyperArc (HA) auto‐planning offers simplicity for the end user and consistently high‐quality SRS plans. The “Ask For It” (AFI) optimization strategy offers a manual planning technique that, when coupled with R50%_Analytic_, can be guided to deliver a plan with an intermediate dose spill “as low as reasonably achievable” and high target dose conformity. A direct comparison of SRS plan quality obtained using the manual planning AFI strategy and HA has been performed.

**Methods:**

Using a CT data set available from the Radiosurgery Society, 54 PTVs were created and used to generate 19 individual SRS/SRT cases. Case complexity ranged from single PTV plans to multiple PTV plans with a single isocenter. PTV locations ranged from relative isolation from critical structures to lesions within 1.5 mm of the optic apparatus and abutting the brainstem. All cases were planned using both the AFI and HA optimization strategies as implemented in the Varian Medical Systems Eclipse Treatment Planning System. A range of treatment plan quality metrics were obtained including Intermediate Dose Spill (R50%), Conformity Indices CI_RTOG_ and CI_Paddick_, PTV Dose Coverage (Dn%), PTV Mean Dose, and Modulation Factor. The Wilcoxon Signed Rank Sum non‐parametric statistical method was utilized to compare the obtained plan quality metrics.

**Results:**

Statistically significant improvements were found for the AFI strategy for metrics R50%, CI_RTOG_, CI_Paddick_, and PTV Mean Dose (*p* < 0.001). HA achieved superior coverage for Dn% (*p* = 0.018), while the Modulation Factors were not significantly different for AFI compared to HA optimization (*p* = 0.13).

**Conclusion:**

This study provides evidence that the AFI manual planning strategy can produce high‐quality planning metrics similar to the HA auto‐planning method.

## 1 INTRODUCTION

Stereotactic radiosurgery (SRS) and stereotactic radiotherapy (SRT) aim to deliver high doses of ionizing radiation to cranial targets in one or a few fractions with high precision. The application of Linac‐based SRS/SRT in the treatment of brain metastases has become increasingly common in radiation therapy clinics.^1^, ^2^, ^3^

Various treatment planning techniques have been employed for the optimization of SRS plans for single isocenter multiple targets (SIMT) SRS^4^, ^5^, ^6^, ^7^; of course, a single target plan is just a simple case of SIMT. A well‐known software‐based, highly automated SRS solution named “HyperArc” (HA) is available for use in the Eclipse Treatment Planning System (TPS) (Varian Medical Systems, Palo Alto, CA). HA is a comprehensive package that includes the creation of the treatment beam geometry, various built‐in treatment planning optimization tools (such as the Collimator Angle Optimizer and predefined SRS Normal Tissue Objective), a collision detection “Virtual Dry Run” module, and integrated automated treatment delivery. The treatment planning component offers a streamlined workflow to consistently produce high‐quality, highly conformal dose distributions.

In a large study at the University of Alabama Birmingham (UAB), Popple and co‐workers have shown that HA provides SRS plans at least as good but often superior to those obtained by expert planners using manual planning methods.^8^ The study from UAB statistically compared several plan quality metrics from a large clinical data set of treatment plans created by in‐house manual planning methods of experienced planners with another large clinical data set of different patients planned using HA.^4^, ^5^ In addition to providing high‐quality SRS plans, the HA approach allows for consistency of plan quality, even among planners of various levels of expertise.

While the HA solution provides an effective means for the delivery of SRS treatments in Radiation Therapy clinics, it is a product limited to implementation on Varian Linacs (Varian Medical Systems, Palo Alto, CA) and the Eclipse TPS planning environment.

The “Ask For It” (AFI) strategy is an SRS optimization methodology shown to produce high‐quality SRS treatment plans for experienced planners.^9^ AFI uses a Dose Volume Histogram (DVH) based optimizer typical in commercially available treatment planning systems. The AFI strategy provides a roadmap for the implementation of an SRS planning solution without the need for specialized vendor‐supplied software. The AFI approach has been successfully utilized for cases involving relatively straightforward, single‐target presentations to more complicated single isocenter multi‐target (SIMT) plans.

This study provides the first direct comparison of the AFI manual planning technique to the HA solution. Identical cases were planned with the AFI strategy and HA to allow an unambiguous comparison of these two optimization approaches.

## 2 METHODS

### 2.1 PTV dataset

To provide a direct comparison of the AFI manual planning technique and the HA auto‐planning solution, a single set of cases for planning that include a CT, Organs At Risk (OAR), and Planning Target Volumes (PTVs) was created; then the identical cases could be planned with both AFI and HA. The Radiosurgery Society (The Radiosurgery Society, San Jose, CA) provided the CT and organs at risk. This high‐resolution CT study contains 417 images with a 1 mm slice spacing and 0.9 × 0.9 mm pixel dimensions. The data contains a structure set of OAR contours. We retained the OAR structure contours but created study‐specific PTVs of various shapes and sizes.

**FIGURE 1:**
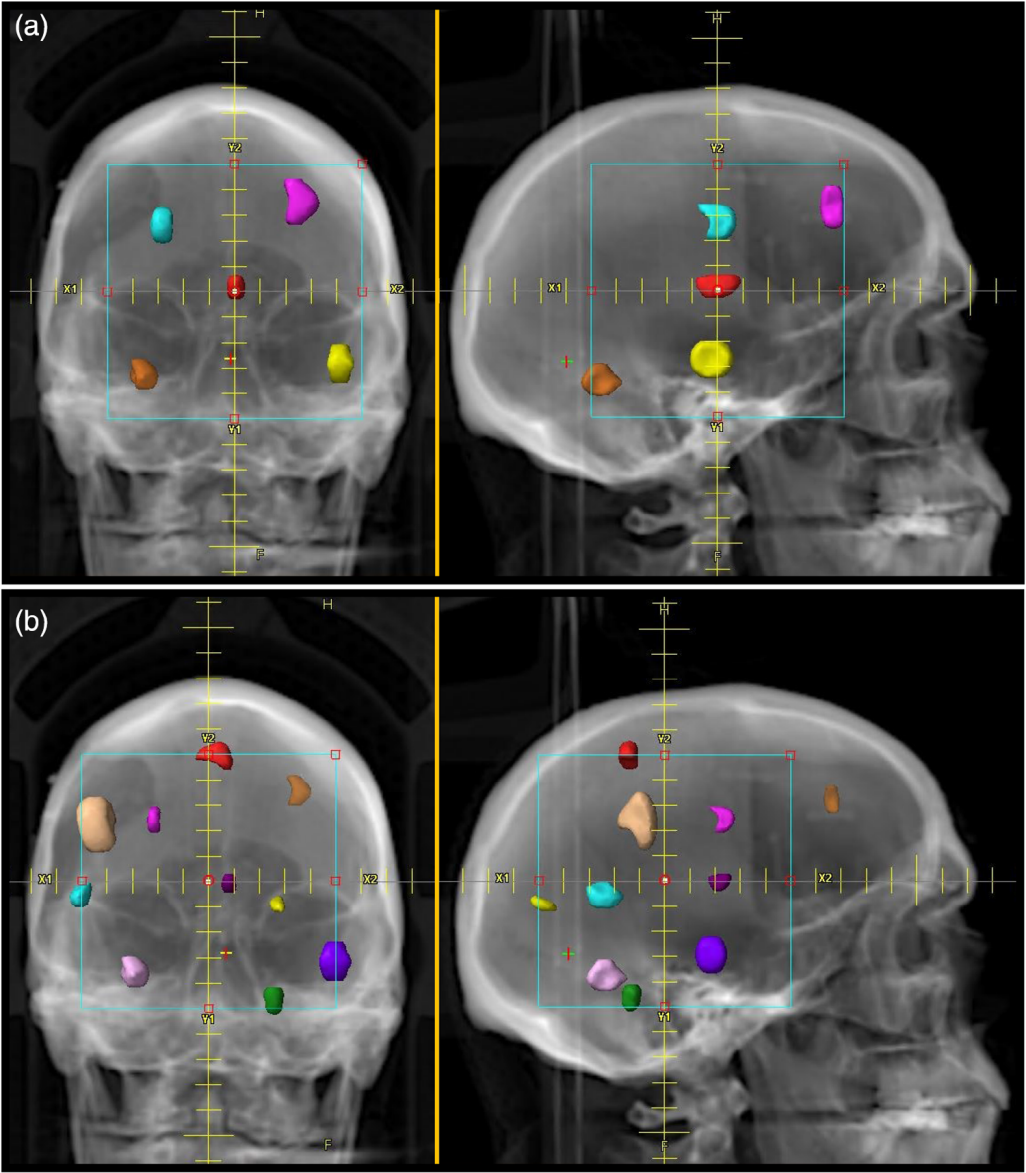
Two representative planning cases with multiple PTVs. (a) Plan 15 contains 5 PTVs (multiple colors). (b) Plan 18 contains 10 PTVs (multiple colors).

**FIGURE 2:**
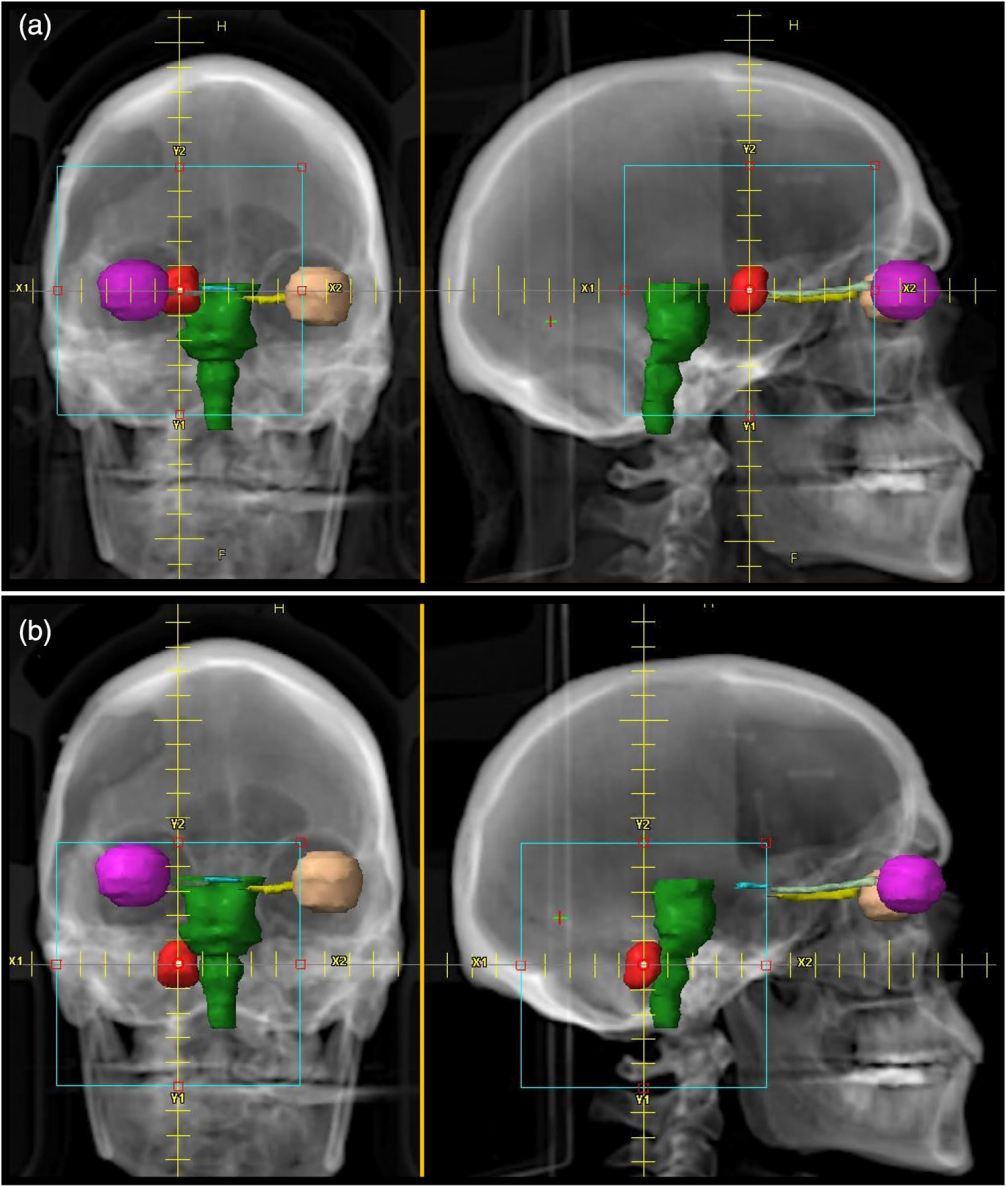
Two representative planning cases involving OARs. (a) Plan 12 contains 1 PTV (red) positioned 1.5 mm from the optic nerve and optic chiasm. (b) Plan 13 contains 1 PTV (red) abutting the brainstem (green).

SRS cases were created with 24 unique PTVs combined in various configurations with differing locations and fractionation schemes for a total of 54 PTVs across 19 individual cases. Each case contains between 1 and 10 PTVs distributed within the cranium at various locations, including abutting the brainstem and within 1.5 mm of the optic chiasm and optic nerve. A mixed set of PTVs was created for a variety of cases including irregular shapes of various volumes ranging from 0.24 to 26.6 cm^3^. The PTVs were created in the contouring workspace within the Eclipse TPS version 16, arbitrarily drawn, but ensuring PTVs are well separated to avoid the complications of overlapping 50% isodose clouds for PTVs that are close together. The PTV shapes were also arbitrary but were specifically non‐spherical. Representative locations of the PTVs for various cases are shown in Figures 1 and 2.

In addition, these cases were assigned a variety of prescriptions including 8 Gy/Fx × 3 Fx, 9 Gy/Fx × 3 Fx, 18 Gy/Fx × 1 Fx, and 21 Gy/Fx × 1 Fx. The specifics of the fractionation were selected to represent a range of typical fractionations. Details of the prescriptions used and the number of PTVs in each case are listed in Table 1. To illustrate the differences between cases, consider the characteristics of Plan 12 and Plan 13 (see Table 1 and Figure 2); the cases share the same single PTV with the same fractionation but at two distinctly different locations. Additionally, consider Plan 18 and Plan 19, which share the same 10 PTVS and locations but were assigned different fractionations. Note that when cases only differ in fractionation, the plans were replanned rather than simply scaled to the new fractionation scheme.

**TABLE 1:** Plan prescription characteristics for the 19 SRS cases used for the AFI – HA comparison.

Plan Index	PTV number	Equivalent PTV diameter (mm)	Plan Rx Dn%	Plan fraction dose (cGy)	Plan no. of fractions
1	1 PTV		D99%	800	3
	1	9.65			
2	1 PTV		D99%	800	3
	1	14.2			
3	1 PTV		D99%	800	3
	1	18.2			
4	1 PTV		D99%	800	3
	1	7.71			
5	1 PTV		D99%	800	3
	1	16.5			
6	1 PTV		D99%	900	3
	1	29.8			
7	1 PTV		D99%	900	3
	1	33.3			
8	1 PTV		D99%	2100	1
	1	7.71			
9	1 PTV		D99%	2100	1
	1	9.65			
10	1 PTV		D99%	2100	1
	1	14.1			
11	1 PTV		D99%	2100	1
	1	18.2			
12^a^	1 PTV		D99%	800	3
	1	18.2			
13^b^	1 PTV		D99%	800	3
	1	18.3			
14	2 PTVs		D95% min	900	3
	1	37.0			
	2	24.9			
15	5 PTVs		D95% min	900	3
	1	13.1			
	2	12.2			
	3	11.0			
	4	12.4			
	5	14.5			
16	5 PTVs		D95% min	1800	1
	1	13.1			
	2	12.2			
	3	11.0			
	4	12.4			
	5	14.5			
17	9 PTVs		D95% min	1800	1
	1	10.4			
	2	8.4			
	3	7.6			
	4	7.3			
	5	10.0			
	6	7.0			
	7	14.1			
	8	12.4			
	9	9.2			
18	10 PTVs		D95% min	900	3
	1	10.4			
	2	8.4			
	3	7.6			
	4	16.0			
	5	7.3			
	6	10.0			
	7	7.0			
	8	14.1			
	9	12.4			
	10	9.2			
19	10 PTVs		D95% min	1800	1
	1	10.4			
	2	8.4			
	3	7.6			
	4	9.65			
	5	14.2			
	6	18.2			
	7	7.71			
	8	16.5			
	9	29.8			
	10	33.3			

*Note*: “Rx Dn%” describes the intended planning goal prescription target coverage. “D99%” is used for single target plans where 99% of the PTV volume receives 100% of the prescription dose. “D95% min” is used for single isocenter multiple target plans and describes the prescription coverage of the least covered PTV. Thus, the minimum dose coverage of the least covered PTV is 95% of the prescription dose. This is a global normalization for the whole plan, and all other PTVs in the plan receive a higher Dn%.

^a^ Plan 12 contains the PTV positioned 1.5 mm from the optic nerve and optic chiasm.

^b^ Plan 13 contains the PTV positioned abutting the brainstem.

The prescription uses the Dn% volumetric nomenclature such that the dose coverage is “*n*%” of the target volume receives the full prescription dose. Thus, D99% means 99% of the target volume receives at minimum the full prescription dose. Single PTV plans are normalized volumetrically to D99%. SIMT plans were normalized to “D95% min,” meaning the minimum coverage of the least covered individual PTV in the multi‐target plan is D95%. “D95% min” is a global normalization within the plan; therefore, the other PTVs in the plan will receive higher prescription dose coverage. The normalization data are listed in Table 1. Since the “least covered PTV” may be a different individual PTV in the corresponding HA plan relative to the AFI plan, the coverage of individual PTVs in a SIMT SRS plan could vary between an HA plan and its corresponding AFI plan. The final achieved normalization data for all PTVs is listed in Table 2.

**TABLE 2:** Plan quality metrics obtained from AFI – HA comparison.

Plan		Achieved Dn%		Equivalent PTV	R50%_Plan_		CI_RTOG_		CI_Paddick_		Mean Dose	
Index	PTV #	AFI	HA	Diameter (mm)	AFI	HA	AFI	HA	AFI	HA	AFI	HA
1	1	99.0	99.0	9.65	4.08	4.31	1.18	1.16	0.83	0.85	116%	114%
2	1	99.0	99.0	14.2	3.09	3.23	1.08	1.07	0.91	0.92	116%	114%
3	1	99.0	99.0	18.2	2.80	2.91	1.07	1.07	0.92	0.91	115%	113%
4	1	99.0	99.0	7.71	4.79	5.42	1.24	1.45	0.79	0.68	116%	120%
5	1	99.0	99.0	16.5	2.86	2.97	1.05	1.04	0.93	0.94	116%	113%
6	1	99.0	99.0	29.8	2.57	2.44	1.01	1.02	0.97	0.96	113%	107%
7	1	99.0	99.0	33.3	2.50	2.35	1.01	1.01	0.97	0.97	114%	106%
8	1	99.0	99.0	7.71	4.75	5.29	1.24	1.28	0.79	0.76	117%	115%
9	1	99.0	99.0	9.65	4.02	4.25	1.14	1.16	0.86	0.85	114%	114%
10	1	99.0	99.0	14.1	3.17	3.25	1.08	1.06	0.91	0.92	115%	115%
11	1	99.0	99.0	18.2	2.82	2.91	1.07	1.08	0.91	0.91	115%	114%
12^a^	1	99.0	99.0	18.2	2.85	2.92	1.06	1.05	0.92	0.93	114%	112%
13^b^	1	99.0	99.0	18.3	2.80	2.89	1.06	1.07	0.93	0.92	115%	113%
14	1	95.0	95.0	37.0	2.22	2.30	0.95	0.97	0.95	0.93	107%	108%
	2	95.0	98.3	24.9	2.56	2.94	0.96	1.05	0.94	0.92	115%	116%
15	1	97.2	97.4	13.1	3.64	3.98	1.02	1.02	0.92	0.93	116%	112%
	2	97.8	97.3	12.2	3.77	4.05	1.04	1.06	0.92	0.90	111%	111%
	3	99.4	95.0	11.0	3.84	4.00	1.09	1.01	0.90	0.90	114%	111%
	4	95.0	96.5	12.4	3.78	4.07	0.99	1.02	0.91	0.92	115%	112%
	5	98.0	96.3	14.5	3.35	3.61	1.04	1.01	0.93	0.92	114%	112%
16	1	97.1	97.2	13.1	3.65	4.00	1.02	1.02	0.92	0.92	115%	112%
	2	98.3	97.2	12.2	3.81	4.07	1.06	1.06	0.91	0.89	112%	111%
	3	99.0	95.0	11.0	3.80	4.04	1.08	1.01	0.91	0.90	114%	111%
	4	95.0	97.0	12.4	3.81	4.08	1.00	1.03	0.90	0.91	115%	112%
	5	98.3	96.2	14.5	3.38	3.66	1.05	1.02	0.92	0.91	114%	112%
17	1	97.5	97.8	10.4	4.41	5.51	1.04	1.06	0.91	0.90	117%	111%
	2	95.0	98.8	8.4	5.35	7.32	1.08	1.28	0.84	0.76	115%	113%
	3	97.3	98.4	7.6	5.87	8.04	1.19	1.29	0.80	0.75	117%	112%
	4	98.1	98.6	7.3	5.15	7.15	1.08	1.19	0.89	0.82	116%	112%
	5	98.3	99.1	10.0	4.43	5.64	1.08	1.12	0.90	0.87	116%	113%
	6	98.2	98.9	7.0	5.83	8.83	1.15	1.21	0.84	0.81	116%	111%
	7	97.5	95.0	14.1	3.32	4.04	1.00	0.98	0.96	0.92	118%	112%
	8	97.2	96.7	12.4	3.70	4.63	1.00	1.02	0.94	0.92	116%	112%
	9	97.9	99.2	9.2	4.46	5.83	1.05	1.07	0.91	0.92	117%	112%
18	1	97.9	98.3	10.4	4.44	5.85	1.06	1.14	0.90	0.85	115%	113%
	2	95.1	98.5	8.4	5.61	7.87	1.08	1.28	0.84	0.76	113%	113%
	3	97.3	98.8	7.6	6.09	8.17	1.19	1.29	0.80	0.76	114%	112%
	4	95.6	95.0	16.0	3.66	4.31	1.00	1.06	0.91	0.85	113%	111%
	5	97.2	98.1	7.3	5.45	7.35	1.07	1.18	0.88	0.81	114%	112%
	6	95.0	99.2	10.0	4.68	5.77	1.01	1.14	0.90	0.86	114%	115%
	7	97.1	99.9	7.0	6.06	8.56	1.08	1.22	0.87	0.82	113%	110%
	8	95.8	98.3	14.1	3.40	4.22	0.97	1.04	0.94	0.93	116%	113%
	9	95.9	98.1	12.4	3.78	4.73	0.98	1.06	0.94	0.91	114%	112%
	10	96.6	99.6	9.2	4.63	6.10	0.99	1.12	0.94	0.89	115%	114%
19	1	98.3	99.2	10.4	4.46	5.75	1.08	1.16	0.89	0.85	115%	114%
	2	95.0	97.4	8.4	5.61	7.52	1.11	1.23	0.81	0.77	114%	114%
	3	97.4	98.0	7.6	6.13	8.13	1.19	1.28	0.80	0.75	116%	112%
	4	96.5	95.0	16.0	3.72	4.30	1.03	1.05	0.91	0.86	113%	111%
	5	97.7	98.5	7.3	5.45	7.15	1.13	1.19	0.85	0.82	115%	112%
	6	97.4	99.1	10.0	4.64	5.26	1.05	1.12	0.90	0.87	115%	114%
	7	97.0	99.4	7.0	6.17	8.39	1.08	1.22	0.87	0.81	114%	111%
	8	96.9	96.1	14.1	3.41	4.20	0.99	1.01	0.95	0.92	117%	113%
	9	97.4	98.1	12.4	3.89	4.60	1.00	1.05	0.94	0.92	115%	112%
	10	97.8	99.6	9.2	4.54	6.12	1.03	1.12	0.93	0.89	116%	113%

*Note*: “Equivalent PTV diameter” is the diameter of an equivalent volume sphere.

^a^ Plan 12 contains the PTV positioned 1.5 mm from the optic nerve and optic chiasm.

^b^ Plan 13 contains the PTV positioned abutting the brainstem.

All plans (HA and AFI) employed 6 MV flattening filter‐free photon (6xFFF) beams and used a standard 4‐arc geometry with an isocenter location determined near the geometric center of combined total PTV (the combination of all individual PTVs within a plan). The treatment couch angle/gantry arc spans are defined as (1) 0°/360°, (2) 45°/180°, (3) 315°/180°, and (4) 90°/180°.

All treatment plans were created and optimized in the same instance of Eclipse TPS using photon optimizer v16 with a final calculation via the AAA v16 algorithm on a 1 mm calculation grid size. All plans were created in Eclipse for the identical linear accelerator, a Varian TrueBeam with 120 leaf HD MLC, using VMAT RapidArc delivery (Varian Medical Systems, Palo Alto, CA).

### 2.2 HyperArc treatment planning

HA is a commercial turn‐key solution for SRS available in the Eclipse TPS that requires specific all‐Varian equipment. The HA solution provides a pre‐defined arc geometry, a HA‐specific SRS Normal Tissue Optimizer (SRS NTO) to control dose conformality, an automatic collimator angle optimizer, and a “Virtual Dry Run” module to assess the potential for gantry collisions with the patient setup. Other planning constraints are at the discretion of the treatment planner.

The HA collimator angles for each plan and individual arcs within the plan are variable and optimized for each case with the HA collimator angle optimizer. The optimization followed the standard HA optimization strategy inherent in the HyperArc product.

### 2.3 AFI treatment planning

AFI is a treatment planning strategy and optimization technique described by Desai et al. that allows one to directly guide the optimizer to deliver a dose that results in the requested R50%.^9^, ^10^ In brief, this strategy uses an optimization shell (OptiForR50%) having a specific design that is custom‐tailored to the characteristics of an individual PTV. AFI also specifies optimization parameters that have been shown highly effective at driving the optimizer toward the desired goals of a treatment plan with a Conformality Index (CI) close to 1.0 and an intermediate dose spill (R50%) “as low as reasonably achievable.” This “as low as reasonably achievable” R50% goal is a theoretical prediction dependent on PTV volume and surface area and is known as R50%_Analytic_.^11^

One minor addition was made to the AFI optimization strategy not described in the previously published literature: a mean dose objective was added to the optimization parameters for the “Brain ‐ (OptiForR50% + PTV)” structure. The OAR structure “Brain ‐ (OptiForR50% + PTV)” is constructed by taking the total brain structure and subtracting all PTV structures and all OptiForR50% structures. The mean dose criteria for “Brain ‐ (OptiForR50% + PTV)” was set to 10% of the prescription dose.

AFI can be used with any delivery geometry (treatment couch angles, arc gantry angle spans, and collimator angles). However, AFI cannot compensate for a vastly inferior beam geometry, and a non‐coplanar 2π beam geometry appears essential for a good result. In this study, we use a well‐established 4‐arc beam geometry used in many SRS planning solutions. As stated earlier, this is the exact 2π geometry also used in the HA approach. The AFI beam geometry could be modified if the planner believes it is warranted, but this was not done in this study.

AFI plans all used collimator angles that were fixed for each couch angle regardless of the PTV configuration for the specific plan. The treatment couch angle/gantry arc span/collimator angle for each VMAT arc is defined as 1) 0°/360°/30°, 2) 45°/180°/45°, 3) 315°/180°/60°, and 4) 270°/180°/315°. HA optimizes a collimator angle for each arc/couch angle combination; however, AFI collimator angles are not case‐specific. Thus, this one‐beam geometry is used for all AFI plans (single PTV and SIMT cases) and has proven to be robust over a large range of clinical presentations when using AFI.

As constructed, the only differences between the HA plans and the AFI plans in this study are collimator angles and optimization strategy. HA uses collimator angles optimized for each plan automatically and the standard HA optimization; AFI uses fixed collimator angles (as discussed above) and the AFI optimization strategy.

### 2.4 Statistical analysis

R Statistical Computing software (The R Project for Statistical Computing version 4.1.3) was utilized to obtain two‐tailed Wilcoxon Signed Rank Sum statistics for PTV plan metrics obtained by either the AFI or HA methodology. Plan quality metrics compared included R50%, CI_RTOG_, CI_Paddick_, PTV Dn%, PTV Mean Dose, and Modulation Factor.

## 3 RESULTS

**FIGURE 3:**
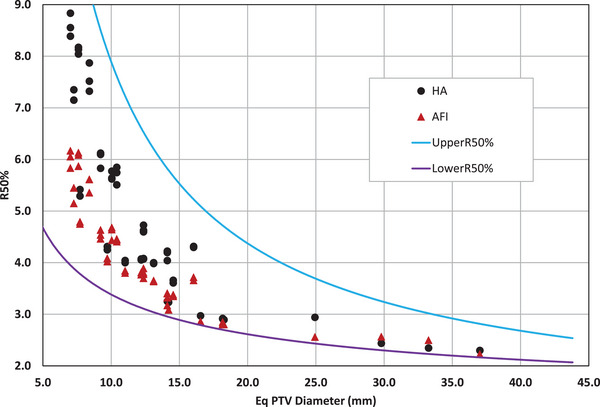
R50% versus equivalent PTV diameter for HA and AFI. HA values are represented with black circles. AFI values are represented with red triangles. The solid lines represent the proposed universal R50% limits, UpperR50% and LowerR50%.^12^

Results from the plan quality metric comparison are summarized in Table 2. The plan quality metric data show a small advantage to AFI over HA for the R50% index in Figure 3. AFI produces plans with lower R50% (intermediate dose spill) with a mean of 4.13 versus the HA R50% mean of 5.02. It is also noteworthy that all R50% values obtained by both AFI and HA are within the clinical spread as shown by UpperR50% and LowerR50%.^12^

**FIGURE 4:**
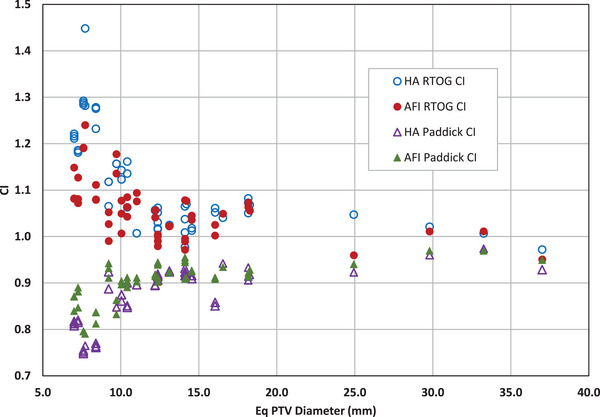
Conformity Index versus equivalent PTV diameter for HA and AFI. HA values are represented with open circles (CI_RTOG_) and open triangles (CI_Paddick_). AFI values are represented with solid circles (CI_RTOG_) and solid triangles (CI_Paddick_).

Similarly, AFI produces comparable conformity indices relative to HA. The CI_RTOG_ mean values for AFI versus HA are 1.06 and 1.11, respectively; the CI_Paddick_ means for AFI versus HA are 0.90 and 0.87, respectively. The slight advantage of AFI over HA for CI_RTOG_ and CI_Paddick_ is shown in Figure 4.

Additionally, the comparison gives comparable results for the PTV dose coverage measures Dn% and Mean Dose. Dn%, the final planning achieved volumetric coverage of each individual PTV, indicates similar target coverage for individual PTVs when comparing AFI and the corresponding HA plans, along with similar Mean Dose for each PTV.

Table 3 summarizes the Modulation Factor (fraction MU/fraction dose), as well as relevant plan data for comparison. In general, the AFI and HA plans often have very comparable total MU and, as a result, similar Modulation Factors (MF). The Modulation Factor means for AFI versus HA are 3.1 and 3.3, respectively.

**TABLE 3:** Modulation factors from AFI – HA comparison.

Plan	No. of	Dose/Fx	Total MU		Mod Factor	
Index	PTVs	(cGy)	AFI	HA	AFI	HA
1	1	800	2423	2354	3.0	2.9
2	1	800	2010	2164	2.5	2.7
3	1	800	1757	1941	2.2	2.4
4	1	800	2391	3105	3.0	3.9
5	1	800	1846	1926	2.3	2.4
6	1	900	1907	2234	2.1	2.5
7	1	900	1910	2247	2.1	2.5
8	1	2100	6287	6013	3.0	2.9
9	1	2100	6108	6051	2.9	2.9
10	1	2100	5141	5650	2.4	2.7
11	1	2100	4581	5091	2.2	2.4
12^a^	1	800	1987	2187	2.5	2.7
13^b^	1	800	1779	1948	2.2	2.4
14	2	900	2594	3229	2.9	3.6
15	5	900	4332	3957	4.8	4.4
16	5	1800	8542	8060	4.7	4.5
17	9	1800	9326	8350	5.2	4.6
18	10	900	4282	4271	4.8	4.7
19	10	1800	8686	8995	4.8	5.0

^a^ Plan 12 contains the PTV positioned 1.5 mm from the optic nerve and optic chiasm.

^b^ Plan 13 contains the PTV positioned abutting the brainstem.

**FIGURE 5:**
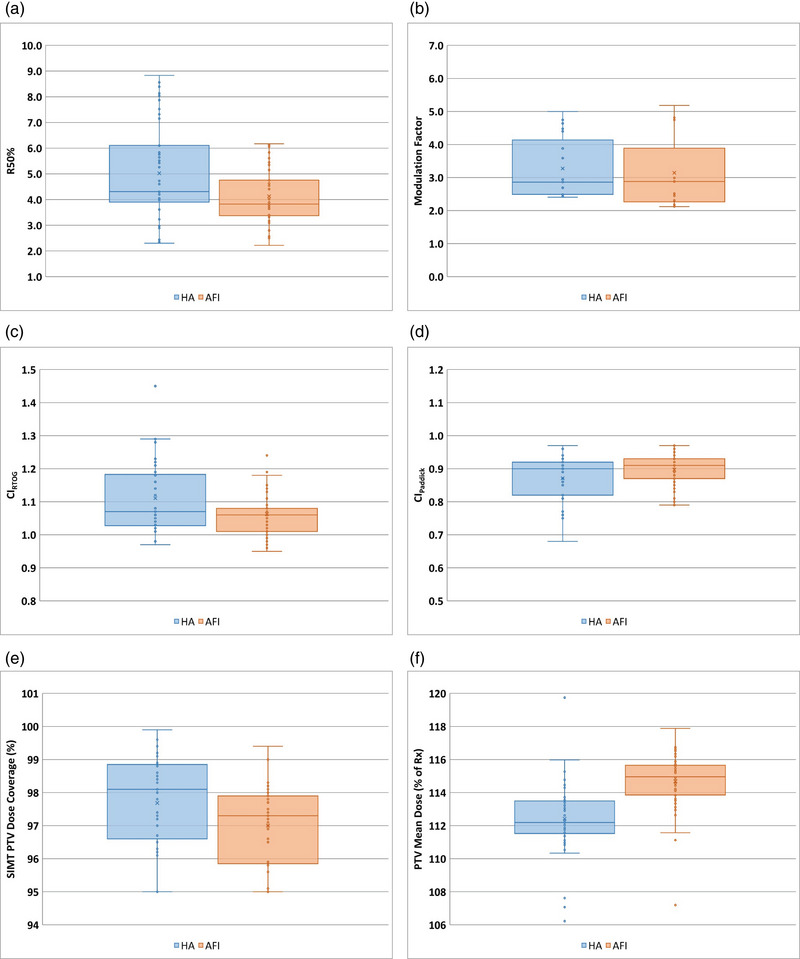
Statistical plots for planning metrics. (a) R50%, (b) Modulation Factor, (c) CI_RTOG_, (d) CI_Paddick_, (e) SIMT PTV dose coverage (Dn%, percent of the PTV volume covered by at least the prescription dose), and (f) PTV mean dose.

Statistical comparisons were performed on the following data sets: 1) R50%, 2) Modulation Factor, 3) CI_RTOG_, 4) CI_Paddick_, 5) SIMT PTV Dose Coverage, and 6) PTV mean Dose. Statistically significant improvement for AFI over HA was indicated for metrics R50%, CI_RTOG_, CI_Paddick_, and PTV Mean Dose (*p* < 0.001), while HA outperformed AFI on PTV Dose Coverage (*p* = 0.018). Modulation Factor differences were insignificant (*p* = 0.13). The mean, median, and quartile range comparisons for these metrics are shown in Figure 5, and a summary of the statistical analyses is provided in Table 4.

**TABLE 4:** Statistical analysis from AFI – HA comparison.

Metric	Statistic	AFI	HA
R50%	Mean	4.13	5.02
	Median	3.83	4.31
	*p*‐value	<0.001	
Modulation factor	Mean	3.1	3.3
	Median	2.9	2.9
	*p*‐value	0.13	
CI_RTOG_	Mean	1.06	1.11
	Median	1.06	1.07
	*p*‐value	<0.001	
CI_Paddick_	Mean	0.90	0.87
	Median	0.91	0.90
	*p*‐value	<0.001	
SIMT PTV dose coverage	Mean	97.0	97.7
	Median	97.3	98.1
	*p*‐value	0.018	
PTV mean dose	Mean	114.7	112.4
	Median	115.0	112.4
	*p*‐value	<0.001	

## 4 DISCUSSION

In a previously published University of Alabama, Birmingham (UAB) study, plan quality metrics obtained from experienced planners using manual planning techniques were compared to results achieved when using the HA auto‐planning solution.^8^ The expansive UAB study compared a cohort of patient cases planned with manual techniques with a different cohort planned with the HA methodology. The study presented here is different than the prior UAB comparison in some fundamental ways and provides a valuable contribution to understanding the effectiveness of the AFI manual planning technique when compared to the commercially available HA auto‐planning platform.

This study is a head‐to‐head comparison as it compares the identical cases planned manually using the AFI strategy and also using HA auto‐planning. The prior comparison of UAB^8^ used a large database of many patients with some cases being planned by UAB's manual planning methods compared to different cases planned with HA. Not having access to such a large cohort of cases, our study relied upon having a smaller set of cases but with each case planned with both methodologies. This head‐to‐head comparison provides a means to directly evaluate AFI versus HA.

The AFI manual planning approach used in this study is well‐defined and could be implemented in a uniform manner among different treatment planners, much like UAB's prescriptive methodology.^8^ The AFI strategy defines an idealized goal for the intermediate dose spill parameter R50%, known as R50%_Analytic_, and a defined set of optimization volumes and constraints unique to each PTV target volume obtainable from this goal R50%_Analytic_.^9^, ^10^ This optimization setup has been scripted and should yield consistent results among planners of varying levels of experience. Therefore, this study can be described as a comparison of results obtained from two different treatment planning approaches, both with well‐defined methodologies, which could be implemented uniformly among different clinics and users.

The R50%, CI_RTOG_, and CI_Paddick_ results are statistically superior for AFI (see Figure 5a,c, and 5d, respectively). The PTV Dn% dose coverage reported in this study shows a slight advantage of HA over AFI. This Dn% analysis only considered multi‐target cases because all single‐target cases are normalized to D99% for both HA and AFI, which should make them automatically identical. HA provided higher coverage of the PTVs with the full prescription dose (see Figure 5e) for these multi‐target cases. AFI provided a higher PTV Mean Dose (see Figure 5f), which might translate into better target ablation.

As indicated in Table 1, the clinical prescriptions for PTV dose coverage (Dn%) are identical for AFI and HA plans. Table 2 indicates the achieved Dn%. Not surprisingly, single target plans are normalized to D99% for both HA and AFI so a comparison of Dn% for these cases is meaningless. However, for the multi‐target cases plan normalization is such that the minimally covered PTV will be characterized by D95%, hence other targets must receive ≥ D95%. The minimally covered PTV will not necessarily be the same target in HA versus AFI. Therefore, it can be meaningful to compare Dn% in multi‐target cases. For example, in Plan Index 18, the minimally covered PTV for AFI is PTV‐6 while the minimally PTV for HA is PTV‐4. This can be explained in part by the differing goals in the optimization approaches. AFI is driven to produce minimal intermediate dose spill as measured by R50% as a main optimization objective. It appears HA drives for better target coverage at the expense of dose conformity.

This in silico study yielded similar Modulation Factors (MF) for AFI and HA plans. The difference in the MF values is not statistically significant (Wilcoxon *p* = 0.13). Considering the plans were designed in the same instance as Eclipse with the same VMAT optimizer for the identical linear accelerator, the similarity of the MFs leads us to expect comparable patient‐specific quality assurance (PSQA) results for the plans, although this remains to be validated.

The only beam geometry difference between the AFI plans and the HA plans is the collimator angle selection. For AFI plans, the collimator angles are always the same for every plan, and all AFI results reported here use the collimator angles specified in Methods, Section 2.3. For the HA plans, the collimator angles are plan‐specific and are determined by the HA collimator angle optimizer. The AFI results with preset collimator angles are comparable to the HA results with optimized collimator angles. This implies a weak dependence on the collimator angle when using AFI optimization for SIMT cases.

The AFI optimization strategy has been proposed as a treatment planning system and Linac‐independent SRS optimization methodology.^9^ While AFI depends only on a DVH‐based optimizer to function, the results could vary from one vendor planning system to another based on differences in the optimization algorithms. This work and the previously published works were all done within the Eclipse planning system, so proving the TPS vendor independence of AFI is an avenue for further investigation.

The AFI strategy provides an effective approach similar to HA in the context of operating a busy clinic. However, the AFI strategy utilizes R50%_Analytic_, which provides an estimate of an idealized minimum value for R50% and allows the planner to gauge the optimality of the plan. With the knowledge of R50%_Analytic_ built into the optimization strategy, one would anticipate R50% values close to R50%_Analytic_. Only one VMAT optimization sequence was performed per plan in this study; however, R50%_Analytic_ can be used as a specific parameter to guide the utilization of additional optimization sequences with modified beam geometry and/or optimization constraints, if needed.

Preliminary tests with inserting the AFI optimization within an HA optimization indicate that using the methods in conjunction may provide HA plans closer to the AFI plans, possibly slightly better (data not shown). This combined approach could lead to improved HA results, but a systematic study would be necessary to establish this. Such an investigation is beyond the scope of this study and could represent an avenue for further exploration.

The AFI optimization strategy can work with any immobilization system; the HA system is currently only functional with the Encompass SRS Fibreplast System (CQ Medical, Avondale, PA). For facilities that own a different SRS immobilization system or have not purchased HyperArc licenses, AFI offers a well‐defined optimization strategy that can yield comparable results.

HA automatically computes many standard plan metrics (CI_RTOG_, CI_Paddick_, GI, but not R50%), whereas the AFI strategy does not directly provide these metrics. One can still calculate the mentioned plan metrics, however, by employing AFI and its optimization rings. By design, AFI's OptiForR50 shell contains the IDC50% and any IDC100% that extends outside the PTV. Thus, the treatment planning system can report the volume of OptiForR50 with a dose ≥ 100% of the prescription and with a dose ≥ 50% of the prescription. These volumes combined with the volume and dose data for the PTV allow for the determination of CI_RTOG_ and R50%. CI_Paddick_ can be determined by (Dn%)^2^/CI_RTOG_, which are all numbers easily obtained from the planning system.^9^

This study compared three plan quality metrics commonly used to assess dose conformality in SRS using a relatively small sample size. The well‐separated PTVs included in the 19 cases of this limited study represent greater than 85% of the PTV size range and complexity of clinical SRS cases.^8^ Abutting and closely‐spaced PTVs were intentionally excluded to avoid the ambiguity of determining individual R50% values for PTVs with overlapping 50% isodose clouds (although a self‐consistent method does exist to quantitatively estimate the R50% even in cases where the IDC50% of multiple PTVs overlap).^13^, ^14^ While these results are encouraging, a larger, more comprehensive study with additional plan quality metrics could provide a more complete assessment of the effectiveness of the AFI methodology.

There are some important implementation details of the AFI approach to note. The AFI plan setup (creating custom‐tailored OptiForR50% shells, entering the optimization parameters, etc.) would need to be done manually by the planner, or each facility would need to create its own script for AFI implementation. As a planning‐only strategy, AFI cannot provide the additional functionality offered by a comprehensive platform such as HA, and the AFI user would assume responsibility for safety issues regarding potential collisions of the gantry with the patient and the correct delivery on the Linac. These are not prohibitive since manually planned and delivered VMAT arc SRS treatments are routinely performed in Radiation Therapy clinics.^4^, ^5^, ^6^, ^8^

While the inherent design of the AFI strategy optimization is independent of a specific treatment planning system (TPS), we are unaware thus far of AFI being tested or implemented outside the Eclipse TPS. AFI defines a unique optimization shell that is custom‐tailored to each PTV and not TPS‐dependent. AFI does require a DVH‐based optimizer to function, but since all commercially available TPS are DVH‐based optimizers, this is not a substantial limitation. Implementing AFI strategy optimization in a non‐Eclipse TPS could represent an opportunity for future study.

It is important to acknowledge that HA is a well‐verified SRS solution that has been clinically proven in many facilities. HA produces and delivers high quality SRS plans with streamlined workflows for planning and delivery. AFI is only a well‐defined optimization strategy and is not currently streamlined into a commercial product with vendor support services that facilitate rapid, safe deployment.

## 5 CONCLUSION

The AFI optimization strategy utilizing R50%_Analytic_ as the R50%_Goal_ yields excellent intermediate dose spill (R50%), as well as comparable CI_RTOG_ and CI_Paddick_, when compared head‐to‐head with HyperArc. AFI coupled with R50%_Analytic_ emulates the function of HA SRS NTO.

## AUTHOR CONTRIBUTIONS

All authors contributed equally to this project.

## CONFLICT OF INTEREST STATEMENT

Dharmin D. Desai is an employee of Varian Medical Systems.

## Data Availability

The data that support the findings of this study are available from the corresponding author upon reasonable request.
